# Co-component signal transduction systems: Fast-evolving virulence regulation cassettes discovered in enteric bacteria

**DOI:** 10.1073/pnas.2203176119

**Published:** 2022-06-01

**Authors:** Lisa N. Kinch, Qian Cong, Jananee Jaishankar, Kim Orth

**Affiliations:** ^a^Department of Molecular Biology, University of Texas Southwestern Medical Center, Dallas, TX 75390;; ^b^HHMI, University of Texas Southwestern Medical Center, Dallas, TX 75390;; ^c^Eugene McDermott Center for Human Growth and Development, University of Texas Southwestern Medical Center, Dallas, TX 75390;; ^d^Department of Biophysics, University of Texas Southwestern Medical Center, Dallas, TX 75390;; ^e^Department of Biochemistry, University of Texas Southwestern Medical Center, Dallas, TX 75390

**Keywords:** co-component signal transduction system, virulence transcription regulation, enteric bacteria, protein structure prediction, protein sequence evolution

## Abstract

Bacterial signal transduction systems sense changes in the environment and transmit these signals to control cellular responses. The simplest one-component signal transduction systems include an input sensor domain and an output response domain encoded in a single protein chain. Alternatively, two-component signal transduction systems transmit signals by phosphorelay between input and output domains from separate proteins. The membrane-tethered periplasmic bile acid sensor that activates the *Vibrio parahaemolyticus* type III secretion system adopts an obligate heterodimer of two proteins encoded by partially overlapping *VtrA* and *VtrC* genes. This co-component signal transduction system binds bile acid using a lipocalin-like domain in VtrC and transmits the signal through the membrane to a cytoplasmic DNA-binding transcription factor in VtrA. Using the domain and operon organization of VtrA/VtrC, we identify a fast-evolving superfamily of co-component systems in enteric bacteria. Accurate machine learning–based fold predictions for the candidate co-components support their homology in the twilight zone of rapidly evolving sequences and provide mechanistic hypotheses about previously unrecognized lipid-sensing functions.

## Significance

Using the domain and operon organization of VtrA/VtrC, combined with fold predictions, we identify co-component signal transduction systems in enteric bacteria that likely regulate virulence. We observe that the heterodimeric VtrA/VtrC periplasmic bile acid receptor controlling the *Vibrio parahaemolyticus* type 3 secretion system 2 is a distant homolog of the ToxR/ToxS master regulator of virulence and has evolved beyond confident sequence recognition. Exploiting the newly developed machine learning methods for structure prediction, we observe a VtrC-like lipocalin fold for both the ToxS periplasmic domain and other detected periplasmic sensor components. This structure prediction supports the divergent evolution of VtrA/VtrC-like co-component signal transduction systems and suggests a role for lipid sensing in regulating virulence in enteric bacteria.

**Fig. 1.**
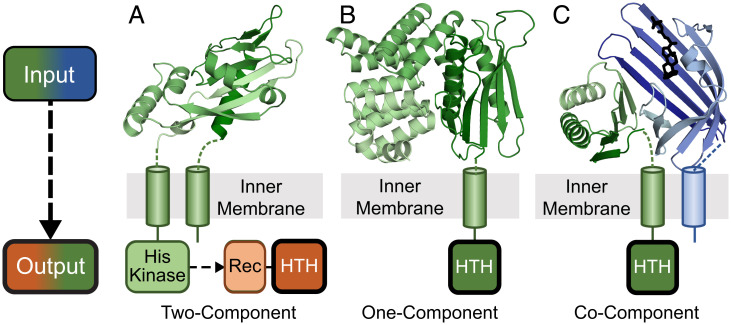
Three transmembrane signaling systems found in enteric bacteria. Extracellular inputs (*Top*, cartoon color scale from dark N terminus to light C terminus) and cytoplasmic outputs (*Bottom*) are depicted for three representative types of bacterial signaling systems. (*A*) Two-component PhoQ (Protein Data Bank (PDB): 1yax) periplasmic input sensor domain (green) is attached through a TMH to an intracellular histidine kinase that signals to a second component, the PhoP output response regulator. (*B*) One-component CadC (PDB: 3ly7) periplasmic domain (green) is attached through a TMH to the output HTH domain. (*C*) Co-component VtrA/VtrC (PDB: 5kew) periplasmic input domains (green/blue), with VtrC attached through a TMH to the output HTH domain.

Bacterial transmembrane  signal transduction systems use periplasmic proteins as inputs for sensing changes in extracellular cues. The input sensors control a variety of adaptive cellular responses by stimulating different intracellular signaling proteins, including chemoreceptors, sensor kinases, and diguanylate cyclases/phosphodiesterases, among others (1). For example, the well-studied Tar and Trg chemoreceptors from *Escherichia coli* transmit environmental signals through a cytoplasmic CheA sensor histidine kinase and CheY receiver as part of a two-component system. These chemoreceptors use a periplasmic ligand binding domain to interact with maltose-, ribose-, and galactose-binding proteins that mediates chemotaxis toward these sugars (2). Often, genes that encode periplasmic solute binding proteins that stimulate sensor histidine kinases are found near their signaling genes (1). Some two-component systems have periplasmic solute binding domains fused directly to their intracellular signaling domains. The virulence regulatory PhoP/PhoQ two-component system includes a PhoQ periplasmic sensor domain connected by a transmembrane helix (TMH) to a cytoplasmic histidine kinase (Fig. 1*A*). Under low periplasmic Mg^2+^ (or Ca^2+^) conditions, PhoQ signals to the receiver domain of its DNA-binding transcriptional regulator PhoP to activate transcription. The *PhoP* and *PhoQ* genes are also neighboring (3, 4).

One-component signal transduction systems typically include an input domain that senses environmental stimuli fused to an output domain that controls an adaptive response. One-component regulators include many of the same input and output domains found in their two-component system counterparts, yet they lack the histidine kinase and receiver (5–7). The diverse domain repertoire of one-component bacterial signal transduction systems most commonly includes various intracellular small molecule-binding input modules that stimulate DNA-binding helix-turn-helix (HTH) output modules to regulate gene expression (6). However, a small subset of one-component transcription factors possesses TMHs and can respond to extracellular stimuli (6, 8, 9). The integral membrane transcription factor CadC exemplifies this type of one-component system, with a periplasmic sensor fused to a cytoplasmic HTH DNA-binding domain (Fig. 1*B*). The periplasmic CadC input module responds directly to low pH to activate the expression of the *CadBA* operon and maintain a neutral cytoplasmic pH. The CadC sensor also binds the feedback inhibitor cadaverine to shut off the system (9–12). The input modules from many of these signaling systems are diverse, lack easily detected sequence similarity to known domains, and can require coregulators for activity (13, 14).

The membrane-tethered ToxR transcription factor that activates the *Vibrio cholerae* virulence regulatory cascade has long been described as a one-component system (15–17). The domain architecture of ToxR includes an N-terminal DNA-binding HTH, a TMH, and a periplasmic domain with a recently determined structure (18, 19). The ToxR HTH belongs to the CadC superfamily of transcription factor DNA-binding domains, while the periplasmic sensor combines an N-terminal αβ-sandwich with a C-terminal α-helical ARM repeat (9, 11). However, the ToxR periplasmic domain structure lacks the CadC sensor subdomain organization and instead requires a second unrelated periplasmic protein ToxS for activity (20). The ToxS coregulator is encoded by a neighboring gene from the ToxR operon (20, 21), and the two gene products form a complex that stabilizes ToxR and renders it resistant to proteolytic cleavage (18, 22). Thus, instead of ToxR serving as a traditionally recognized one-component system, we suggest that the periplasmic ToxR/ToxS heterodimer serves as a sensor in a “co-component” signal transduction system. The ToxR/ToxS sensor responds to various environmental cues, such as bile salts, pH, and redox state (18, 19, 23–25), although the molecular mechanism of activation by these factors is not fully understood.

The ToxR CadC-like HTH family includes another membrane-tethered transcription factor (VtrA) from *Vibrio parahaemolyticus*. VtrA possesses a similar domain composition as ToxR and functions together with a neighboring genomic coregulator (VtrC) (8). VtrA/VtrC serves as a bile salt sensor that ultimately activates the transcription of the type III secretion system 2 (T3SS2) virulence system during *V. parahaemolyticus* infection of the human gut (13, 26). The periplasmic VtrA/VtrC structure adopts an obligate heterodimer that binds bile acid using a lipid-binding lipocalin-like fold from VtrC (13). Despite a significant divergence between the VtrA and ToxR periplasmic sequence, the two adopt a similar fold that defines the VtrA/ToxR periplasmic input modulator superfamily (13, 19). Given the fast-evolving nature of this input modulator superfamily, we suspect that the corresponding ToxS could also be homologous to VtrC and adopt a lipocalin-like fold.

Protein folds can often be inferred from their evolutionary relationships to experimentally determined structures. These relationships have traditionally been detected at the sequence level by homology-based protein structure prediction methods. However, for fast-evolving domains such as in the periplasmic regions of VtrA/VtrC and ToxR/ToxS, identifying homologs using sequences has proven difficult, even for the most sensitive methods (27–29). Here, we take advantage of the VtrA/ToxR transmembrane-containing domain organization and its preserved neighboring gene arrangement to identify candidate co-component systems in enteric bacteria that have evolved beyond sequence recognition. The structures of similar fast-evolving sequences were recently predicted with an accuracy approaching that of experimental structures by machine learning (30–33). Using these structure prediction methods (31, 32, 34), we provide evidence that the identified candidates adopt VtrA/VtrC-like folds. The fold predictions suggest that the two-gene cassettes encoding co-component signaling systems evolved from a common ancestor and provide functional implications for the new superfamily members (Fig. 1*C*). This example highlights the role of artificial intelligence–based structure prediction in shifting a structure biology paradigm, where de novo predictions can now inform homology beyond sequence detection limits.

## Results

### The *ToxR/ToxS* Gene Cassette Encodes Fast-Evolving Homologs of VtrA/VtrC.

**Fig. 2.**
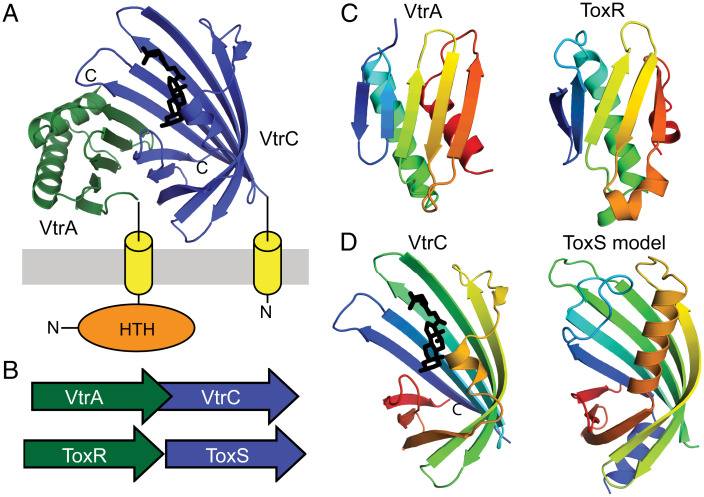
VtrA/VtrC T3SS2 virulence regulon is homologous to ToxR/ToxS. (*A*) The VtrA/VtrC structure (PDB: 5kew) depicted in cartoon adopts an obligate heterodimer of the periplasmic VtrA (green) and VtrC (blue) that transmits an input signal from VtrC-bound bile acid (black stick) through the membrane (gray) via the TMH (yellow cylinder) attached to the N-terminal VtrA DNA-binding HTH (orange sphere). (*B*) The *VtrC* gene (green arrow) encoding the coregulator is downstream from the *VtrA* gene (blue arrow) encoding the transcription factor in an overlapping operon that is arranged similarly to the genes of the *ToxR* (green arrow) and *ToxS* (blue arrow) operon. (*C*) The labeled VtrA periplasmic domain, colored in rainbow from the N terminus (blue) to the C terminus (red), adopts the same fold as the labeled ToxR periplasmic domain (PDB: 6utc). (*D*) The labeled VtrC periplasmic domain that binds the bile acid TDC (black stick) adopts the same fold as the predicted ToxS structure model.

The periplasmic VtrA/VtrC structure adopts an obligate heterodimer that senses the bile acid taurodeoxycholate (TDC) using a lipocalin-like fold adopted by the VtrC component (Fig. 2*A*). The bile acid input transduces a signal through the membrane to the VtrA HTH DNA-binding domain and initiates transcription of the *V. parahaemolyticus* T3SS2 virulence secretion machinery. The two genes encoding the VtrA/VtrC co-component signal transduction system are neighboring in the *V. parahaemolyticus* genome and form an operon (Fig. 2*B*). Functional similarities between the VtrA/VtrC and ToxR/ToxS systems and their preserved operon arrangement (Fig. 2*B*) suggest that their homologous DNA-binding output domain relationship could extend across the whole two-gene cassette. Accordingly, the periplasmic folds of VtrA and ToxR are related, adopting a similar αβ–sandwich fold (Fig. 2*C*). Despite this structure similarity, sensitive sequence detection methods starting from either of the periplasmic domain sequences confidently identify sequences from the same family, but not across families (*SI Appendix*, Table S1). Thus, VtrA and ToxR exemplify homologous transcription factors whose periplasmic input domains have evolved beyond sequence recognition.

The rapidly diverging VtrA and ToxR periplasmic domains interact with their cotranscribed operon partners VtrC and ToxS, respectively. The VtrC structure adopts a lipocalin-like fold that typically binds lipid (Fig. 2*D*). However, the structure of the corresponding ToxR operon partner ToxS is unknown, and sensitive sequence detection methods fail to identify a relationship between ToxS and VtrC (*SI Appendix*, Table S1). Despite this lack of a detectable sequence relationship, the ToxS sequence does identify an alternate lipocalin-like fold with borderline confidence and coverage (*SI Appendix*, Table S1). This hint at ToxS adopting a similar fold as its VtrC counterpart prompted us to build a structure model using newly developed structure prediction methods, including AlphaFold and ColabFold (31, 32, 34, 35). Indeed, the ToxS model adopts a lipocalin-like fold similar to VtrC (Fig. 2*D*). High confidence is assigned to all residues that constitute the core lipocalin-like domain. Together with the similar domain organization and periplasmic folds of ToxR and VtrA, this ToxS structure prediction supports the proposed homologous relationship between the *ToxR/ToxS* and *VtrA/VtrC* gene cassettes.

### Genome Neighborhoods Facilitate Discovery of Candidate VtrA/VtrC-Like Co-component Systems.

Given the divergent nature of the VtrA/VtrC periplasmic input components, we reasoned that additional membrane-tethered co-components might exist. Accordingly, sequence search using the VtrC periplasmic domain identified the PsaF superfamily with borderline confidence (*SI Appendix*, Table S1). PsaF works together with its neighboring membrane-tethered transcription factor PsaE to control the expression of the pH6 antigen adhesin virulence factor in *Yersinia* (36). The PsaE DNA-binding domain is related to both ToxR and another positive regulator of *V. cholerae* virulence, TcpP, which works together with its neighboring TcpH periplasmic input domain (37). Each of the transcription factors has the same domain organization, with an N-terminal HTH domain, followed by a single TMH, and a C-terminal periplasmic domain of unknown structure. Thus, the similar domain organization, transcription regulatory function, and neighboring gene organization suggest that the VtrA/VtrC-like superfamily could exist as a mobile two-gene cassette that controls virulence in *Vibrio* as well as in other enteric pathogens.

While the periplasmic components of VtrA/VtrC, ToxR/ToxS, and PsaE/PsaF have diverged beyond confident sequence recognition, the CadC-like HTH retains significant sequence similarity and can be used to search for candidate co-component systems in other pathogenic bacteria. Starting from VtrA and ToxR, we searched a database of well-studied bacterial pathogens with complete genomes of high sequence and annotation quality (18 genomes; *SI Appendix*, Table S2). The VtrA/ToxR sequence search identified numerous CadC-like HTH-containing proteins from all represented genomes (252 sequences total). These identified sequences were filtered using TMH prediction, resulting in 28 transmembrane-tethered transcription factors from eight species of enterobacteria. To further filter these identified transcription factors for candidate VtrA/VtrC-like co-component signal transduction systems, we inspected their gene neighborhood for a similar potential cotranslated operon organization. Adjacent downstream genes in the same orientation as the identified transcription factors were subjected to transmembrane and signal peptide prediction. One notable candidate co-component system from *Yersinia pseudotuberculosis* required an N-terminal extension of the predicted start site to include the TMH (BZ17_3565; for simplicity, “BZ17_” is replaced with “YP” in the subsequent text). This alternate start site was likely misannotated due to its reading frame overlap with the upstream transcription factor (as was seen with the *VtrA/VtrC* genes) and is extended correctly in close orthologs from other *Y. pseudotuberculosis* strains.

Inspection of genomic neighborhoods excluded membrane-tethered CadC one-component transcription factors, for which the VtrA HTH domain is named, and dismissed other pairs that lack input domains or neighboring proteins predicted to be in the periplasm. In addition to the known *Vibrio ToxR/ToxS*, *VtrA/VtrC*, and *TcpP/TcpH* gene cassettes, several gene pairs from other enteric bacteria passed the search criteria (Table 1). *Y. pseudotuberculosis* includes two novel gene cassette pairs of unknown function in addition to *TcpP/TcpH*. *Shigella flexneri* encodes a single candidate co-component pair of unknown function, while *Salmonella typhimurium* encodes three candidate pairs. Two gene cassette pairs are neighboring in the genome (*STM0341*/*STM0342* and *STM0344*/*STM0345*), while the last represents the *MarT* (38)/*FidL* cassette from pathogenicity island 3 (Spi-3). The *MarT/FidL* gene products are cotranscribed and activate expression of the MisL autotransporter, which functions as a host-specific intestinal colonization factor (38–40). The noninvasive *E. coli* K12 strain includes a single candidate cassette, *YqeI/YqeJ.* The *YqeI* and *YqeJ* genes are described as a remnant of the ETT2 (type III secretion system) pathogenicity island in K12 that is fully present in pathogenic strains like *E. coli* O157:H7 saki, which also encodes YqeI/YqeJ. In addition to this virulence regulator, the pathogenic *E. coli* O157:H7 saki strain encodes an additional cassette, *GrvA* (41)/*FidL*. The GrvA transcription factor activates the locus of enterocyte effacement (LEE)–dependent adherence.

**Table 1.** Candidate co-component systems

Bacteria	ToxR ORF; Gene	Neighbor	Comment
*V. parahaemolyticus*	*VP0820; ToxR*	*ToxS*	ToxS lipocalin
*V. parahaemolyticus*	*VPA1332; VtrA*	*VtrC* *	Known
*V. cholerae*	*MS6_0632; TcpP*	*MS6_0633* *	TcpH lipocalin
*V. cholerae*	*MS6_0778; ToxR*	*MS6_0777*	ToxS lipocalin
*Y. pseudotuberculosis*	*BZ17_3564*	*BZ17_3565* *	Lipocalin
*Y. pseudotuberculosis*	*BZ17_1189; PsaE*	*PsaF* *	PsaF lipocalin
*Y. pseudotuberculosis*	*BZ17_3283*	*BZ17_3282* *	Lipocalin
*E. coli* K-12	*ECK2845; YqeI*	*ECK2846*	YqeJ lipocalin
*E. coli* Sakai	*ECs_3704; YqeI*	*ECs_3705*	YqeJ lipocalin
*E. coli* Sakai	*ECs_1274; GrvA*	*ECs_1273*	FidL lipocalin
*S. typhimurium*	*STM3759; MarT*	*STM3758* *	FidL lipocalin
*S. typhimurium*	*STM0341*	*STM0342* *	Half barrel
*S. typhimurium*	*STM0344*	*STM0345* *	Lipocalin
*S. flexneri*	*SF3508*	*SF3507* *	Lipocalin

*Overlapping open reading frame.

### VtrC Defines a Fast-Evolving Lipocalin-Like Sensor Superfamily.

**Fig. 3.**
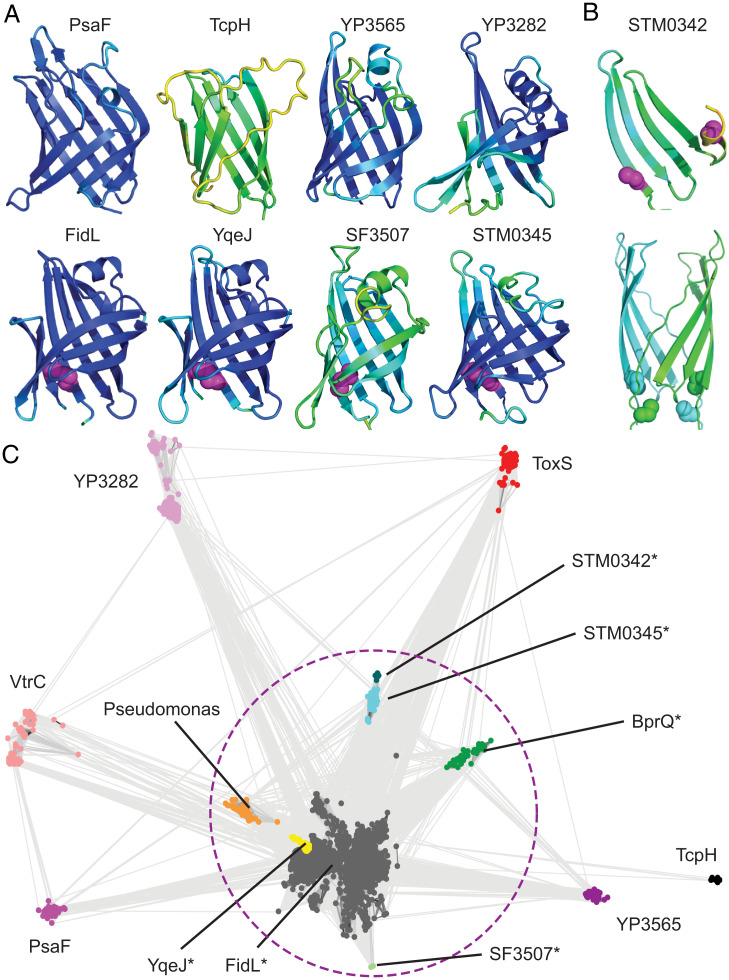
Lipocalin-like folds unite diverse VtrC-like members. (*A*) Models for VtrC-like representatives are colored in rainbow scale from blue (highest confidence) to red (lowest confidence; note the confidence is too high to see any red), with Cys disulfides in magenta sphere. (*B*) STM0342 monomer (*Top*, depicted as in *A*) and dimer model (*Bottom*, cyan and green) with Cys residues (sphere). (*C*) VtrC-like sequences (nodes, colored according to family and labeled) are clustered with CLANS in two dimensions. Connecting lines denote similarity between nodes (< 0.0001 BLAST E-value cutoff). Families marked with (*) can be linked by sequence using PSI-BLAST and are circled with a dotted magenta line.

The candidate co-component system gene cassettes include VtrA-like transcription factors and their adjacent periplasmic components. However, the adjacent periplasmic components tend to lack sequence similarity to known domains (42, 43). Given the ability of AlphaFold to confidently predict the fast-evolving ToxS structure, we generated models for each candidate VtrC-like co-component. For all but one case, the modeled structure adopted a lipocalin-like fold with eight meandering β-strands forming a VtrC-like barrel (Fig. 3*A*). Like the ToxS prediction (Fig. 2*D*), most of the models have high estimated confidence for residues in the core fold. However, the *Shigella* unknown protein SF3507 and the *V. cholerae* TcpH models were less-confident predictions. The final candidate gene cassette, STM0342, was predicted as a four-stranded β-meander that corresponds to half of the barrel and may represent a deterioration of its sequence-related neighbor STM0345. Interestingly, STM0342 includes two Cys residues located too far from one another to form an intrachain disulfide, and the deterioration may form a homodimer suggested by an AlphaFold model (Fig. 3*B*).

The lipocalin-like structure predictions for each of the VtrC-like components support the notion that the second periplasmic component from each gene cassette arose from a common ancestor, despite their sequence divergence. In support of this proposed homologous relationship, the lipocalin-like folds from the *E. coli*, *Shigella*, and *Salmonella* structure models possess a conserved disulfide formed between cysteine residues from the adjacent N-terminal and C-terminal strands of the barrel (Fig. 3*A*, magenta spheres), and the sequence relationship between *Salmonella* Spi-3 (*MarT/FidL*) and *E. coli* ETT2 (*YqeI/YqeJ*) has been noted (44). The presence of these conserved disulfides suggests that some of the newly identified VtrC-like superfamily members may retain enough of a sequence signal to be recognized. To understand the sequence relationships between the identified families, we collected homologs for VtrC-like representatives and used these to build sequence profiles for each family. Profile comparisons using HHsearch (*SI Appendix*, Table S3) highlight the relationships between family members, where the disulfide-containing proteins FidL, YqeJ, STM0342, STM0345, and SF3507 are confidently related by sequence. The remaining families have diverged beyond confident sequence recognition using the most sensitive sequence comparison methods (HHsearch; *SI Appendix*, Table S3).

To visualize the relationships between all the VtrC-like families, collected sequence homologs were clustered (Fig. 3*C*). The disulfide-containing FidL-like sequences (Fig. 3*C*, gray nodes) overlap with the YqeJ-like sequences (Fig. 3*C*, yellow nodes) and are difficult to distinguish. Alternatively, the clusters for the other confidently related disulfide-containing families (i.e., SF3507, STM0342, and STM0345; Fig. 3*C*, enclosed by a dashed circle) are more separated. These diverging sequence-related clusters provide further support for the fast evolution of the superfamily. The cluster sizes (and distances from large clusters) of the various superfamily members tend to mimic the confidence values associated with the AlphaFold models, with TcpH (a singleton) being the least confident prediction. Thus, the presence of such sparsely populated and fast-evolving families among the VtrC-like periplasmic components lacking the conserved disulfide has likely prevented their identification by traditional sequence-based methods.

Two additional groups of collected homologs diverge from the FidL cluster, including BprQ-like sequences and sequences from various *Pseudomonas* species (Fig. 3*C*, green nodes and orange nodes, respectively). The BprQ cluster includes a T3SS3 regulator from the Melioidosis pathogen *Burkholderia pseudomallei*, which has recently been shown to be an enteric bacterium (45). The BprQ sequence adopts a predicted lipocalin-like fold with the preserved FidL-like disulfide. The *BprQ* gene is adjacent to *BprP*, which encodes a transmembrane-anchored transcription factor. BprP/BprQ regulates the machinery and secretion components of the *B. pseudomallei* T3SS3 (46, 47). BprP retains the same domain organization as other VtrA superfamily transcription factors, with an N-terminal HTH followed by a TMH and a periplasmic region with the same predicted fold as the other superfamily members. The second detached cluster includes sequences from various other *Pseudomonas* strains, although the *Pseudomonas aeruginosa* PAO1 representative genome (*SI Appendix*, Table S2) lacks candidate co-component systems. One of the VtrC-like sequences from *Pseudomonas fluorescens* strain C1 overlaps with a VtrA-like transcription factor, highlighting the preserved operon organization in this species.

### Functional Implications for the VtrA/VtrC-Like Co-component Gene Cassettes.

**Fig. 4.**
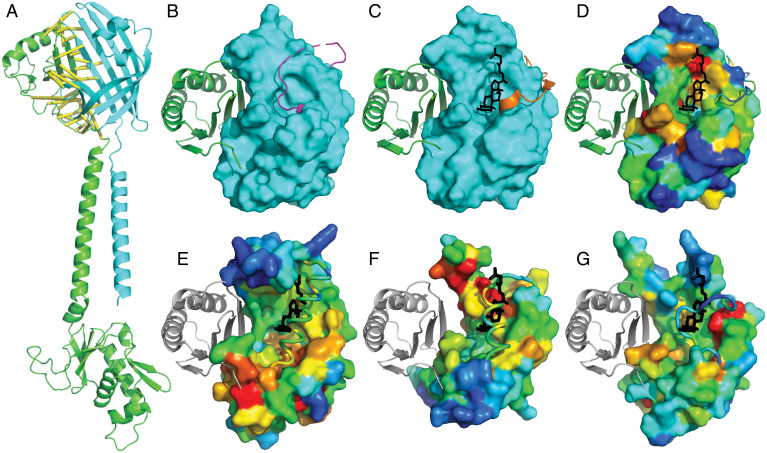
Functional implications of VtrA/VtrC superfamily fold prediction. (*A*) GrvA (green cartoon) complex model with FidL (cyan cartoon). Confidently predicted residue–residue contacts are connected by yellow bars (residue–residue distance in structure model ≤ 8 Å and predicted aligned error ≤ 4 Å). (*B*) VtrA (green cartoon) experimental structure bound to ligand-free VtrC (cyan surface), with a mobile loop (magenta) covering the lipid binding site. (*C*) VtrA/VtrC experimental structure bound to TDC (black stick) displaces the mobile loop (orange). (*D*) VtrC surface is colored in rainbow by family-based residue conservation from low (blue) to high (red). (*E*) ToxS, (*F*) YP3282, and (*G*) BprQ structure models (rainbow conservation) are superimposed with VtrC to highlight relative positions of VtrA (gray cartoon) and TDC (black stick). The corresponding mobile loops (cartoon tubes) cover the potential lipid binding sites as in the apo VtrA/VtrC structure.

The VtrA/VtrC periplasmic bile receptor forms an obligate heterodimer, and many of the identified candidate co-components are known to function together (13). Because protein–protein complex structure predictions can now be generated with increasing accuracy for larger families (31, 48, 49), we reasoned that the sequence information from the larger FidL cluster (Fig. 3*B*) would be adequate for predicting its interaction with GrvA. Indeed, the structure model of the GrvA transcription factor in complex with FidL retains a similar interaction surface as in the VtrA/VtrC experimental structure, with the top predicted residue–residue contacts lining the periplasmic domain interaction surface (Fig. 4*A*, yellow lines). The same β-barrel extension of a split observed in VtrC by the VtrA periplasmic β-sheet (Fig. 2*A*) is present in the FidL/GrvA complex (Fig. 4*A*). The TMH and relative position of the GrvA HTH are less confidently placed based on the predicted aligned error, and the per-residue confidence metric reflects lower accuracy in the TMH of both components as well as in the GrvA-connecting helix and longer HTH domain loops (*SI Appendix*, Fig. S1). Protein–protein complex prediction for many of the other candidate co-components suggests similar interactions, although with generally lower confidence. In support of their heterodimeric interaction, the periplasmic domains from the co-component signal transduction systems PsaE/PsaF from *Yersinia* and YqeI/YqeJ from *E. coli* form dimeric complexes when expressed together (*SI Appendix*, *Methods*).

The VtrA/VtrC periplasmic structure forms an obligate heterodimer with a mobile loop covering the lipid binding site in the absence of bile acid (Fig. 4*B*). Upon binding the bile acid TDC, the mobile loop opens to adopt an alternate conformation (Fig. 4*C*). Like other lipocalin folds (13), VtrC binds TDC on one side of the barrel center. This preserved binding mode, together with the lipocalin-like fold predictions of the other VtrC-like components, suggest that they may also function as lipid sensors. Such functional sites are often conserved, and the residues that contribute to the binding site can be identified using multiple sequence alignments of family members. As a proof of this concept, conserved residue positions from VtrC contribute to the TDC binding site, and the VtrA interaction surface is also relatively conserved (Fig. 4*D*).

To gauge whether the newly identified periplasmic components might bind lipids, we defined per-residue conservations for several of the VtrC-like clustered sequence groups and mapped them to the structure models to highlight potential functional surfaces. Superposition of the ToxS structure model with the experimental VtrC model bound to TDC (Fig. 4*E*) highlights a loop in ToxS that covers the potential lipid binding site. The loop corresponds to the mobile loop in VtrC, and it positions a helix in the corresponding lipid site. The covered lipid binding site is generally more conserved in ToxS than the surrounding surface, with the most conserved region surrounding the interior part of the cleft located deeper in the barrel. Portions of a potential ToxR binding site are represented by the superimposed VtrA periplasmic domain (Fig. 4*E*, white cartoon) and are particularly conserved at the surface interacting with the VtrA N-terminal loop. This conserved surface in ToxS may help position the ToxR TMH (connected to the loop), which could ultimately control the position of the intracellular DNA-binding domain.

Conservations in the YP3282 structure model (Fig. 4*F*) surround the exterior portion of the potential lipid binding cleft, which is also covered by a helix in the mobile loop. As opposed to the conservations in ToxS, the most conserved portion of the potential YP3282 transcription factor periplasmic domain surface lies on the sheet opposite to the N-terminal loop. Conservations in the BprQ structure model (Fig. 4*G*) also mark a potential lipid binding site that is covered by the mobile loop. However, the loop in BprQ is shorter and lacks a helix. Like the surface conservations in ToxS, the presumed BprP periplasmic domain binding site is most conserved near the N-terminal loop. Thus, while the various family members generally preserve conservations in the potential lipid binding site and the co-component interaction surface, these conservations differ between divergent members of the larger superfamily.

### Relationship Between the VtrA-Like Co-components and CadC.

**Fig. 5.**
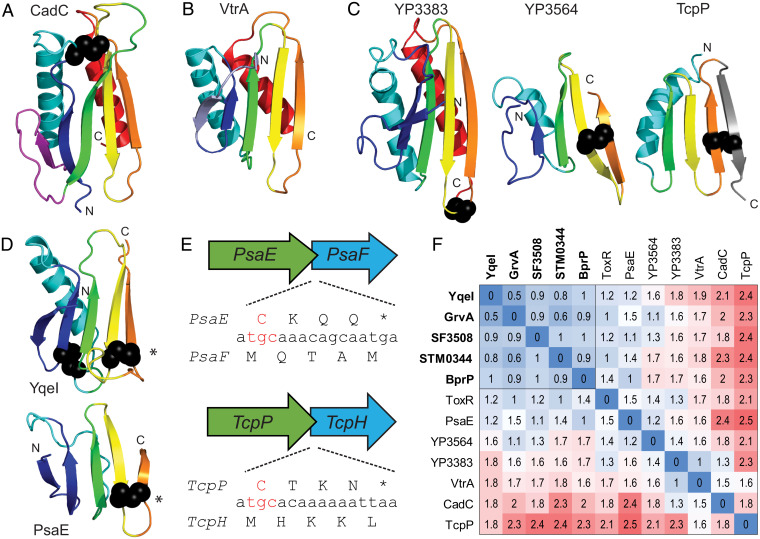
VtrA periplasmic domain evolution. (*A–D*) Periplasmic domain SSEs are in rainbow cartoon from the N terminus (blue) to the C terminus (red), with disulfides in black sphere. (*A*) CadC periplasmic domain has an insertion (magenta) with respect to (*B*) the VtrA periplasmic domain, with the CadC insertion replaced by an N-terminal extension (slate). (*C*) Candidate co-component VtrA-like models are colored by the core SSEs in CadC and VtrA. (*D*) A conserved disulfide (*) links YqeI and TcpP, which includes a C-terminal extension (gray). (*E*) Representative operon ORFs (labeled by gene) with conserved C-terminal Cys codon (red) overlapping with start codon from input component. (*F*) Distance matrix of all-against-all structure comparisons colored by distance in red (distant)-white-blue (identical) color scale. Components that interact with confident sequence-related VtrC-like domains are bolded, and their distances are boxed.

The membrane-embedded one-component transcription factor CadC includes an N-terminal HTH, followed by a TMH and a C-terminal periplasmic sensor (Fig. 1*B*). This domain organization is retained in the DNA-binding chain of the co-component systems (Fig. 1*C*). These similarities could suggest that the CadC and VtrA-like periplasmic domains arose by divergence from a common ancestor. The CadC one-component periplasmic sensor includes two domains. The first adopts an αβ-sandwich, and the second adopts an α-helical domain of tetratricopeptide repeats (Fig. 1*B*). The structure of the CadC αβ-sandwich (Fig. 5*A*) includes an N-terminal α/β/α unit with a Rossmann-like crossover, followed by a three-stranded β-meander and a C-terminal α-helix. If the β-strand and loop from the Rossmann-like crossover represents an insert, then the CadC periplasmic sandwich retains the same core set of secondary structure elements as in the VtrA co-component periplasmic domain (Fig. 5*B*), with an N-terminal β-strand extension in VtrA replacing the Rossmann-like insertion strand from CadC. Superposition of the two domains results in structure similarity (Dali Z-score 5.6) over the βαβ (3)α core that is above the suggested cutoff for significance (Z-score 2) and is higher than the corresponding structure similarity between the related ToxR and VtrA periplasmic domains (Z-score 4.1). However, these scores reside within the “gray area” of homology, and many existing structures exhibit higher similarities to the CadC fold, as VtrA is ranked 34th in a search against a nonredundant structure database (50, 51).

The lack of sequence similarity combined with marginal structure similarity between the CadC and VtrA periplasmic domains precludes sequence-based clustering or traditional phylogenetic analysis to evaluate evolutionary relationships. However, the models for the VtrA-like periplasmic domains allow a quantitative comparison of the structures as well as a definition of the conserved secondary structure elements (SSEs) that contribute to the fold. The experimental VtrA and ToxR periplasmic domain structures include a common set of SSEs (β (2)αβ (3)α) that can be compared to the rest of the VtrA-like components (Fig. 5 *C* and *D*). The *Yersinia* YP3564 model is the only one with the C-terminal helix found in VtrA and ToxR, while its distantly related paralog (YP3383) model lacks the helix and shows a deteriorating N-terminal strand. The models for several of the VtrA-like components (YqeI, GrvA, SF3508, STM0344, and BprP) retain a similar overall fold without the C-terminal helix (YqeI in Fig. 5*D*). The N-terminal helix has also deteriorated into a connecting loop in the PsaE model (Fig. 5*D*). Finally, the model for TcpH lacks both N-terminal β-strands and gains a C-terminal β-strand. The interaction mode for the VtrA/VtrC co-component system uses the C-terminal strand to extend a sheet in the opened VtrC barrel. The lack of a C-terminal helix would better expose the C-terminal edge strand for such an interaction (as in Figs. 2*A* and 4*A*), and the deterioration of SSEs on the opposite side is consistent with their distance from the functional interaction surface. However, the C-terminal extension of the sheet in TcpP would not be compatible with the VtrA/VtrC-like interaction.

Notably, except for VtrA, the co-component transcription factor periplasmic domains contain conserved disulfides. Each of these includes a Cys residue near the C terminus of the protein sequence (Fig. 5 *B* and *D*). With a few exceptions (ToxR/ToxS, YqeI/YqeJ, and GrvA/FidL), the transcription factor periplasmic domain C terminus overlaps with the N terminus of the neighboring co-component gene, which is encoded by a frame shift (Fig. 5*E* and *SI Appendix*, Fig. S3). The starting Met (atg codon) from the downstream gene tends to overlap with the Cys (tg(t/c) codon) from the disulfide. In this case, the starting Met from the second gene is shifted by one nucleotide upstream with respect to the first gene. The *TcpP/TcpH*, *PsaE/PsaF*, *YP3564/YP3565*, *STM0341/STM0342*, and *STM0344/STM0345* gene overlaps observe this tendency. Alternatively, *SF3508*/*SF3507* and *YP3283*/*YP3282*, which are encoded in the complementary strands, include the Cys residues in their overlap. Finally, the MarT/FidL overlap includes an His residue (cat codon) shifted one nucleotide upstream with respect to the MarT overlapping Met (atg codon).

A heat map of distances calculated from pairwise comparisons of the VtrA-like periplasmic domain structures highlights their relationships (Fig. 5*F*). A subset of VtrA-like periplasmic domains (YqeI, GrvA, STM0344, SF3808, and BprP) that interact with the sequence-related VtrC-like components retain relatively lower distances. These domains include a conserved disulfide bond between the last two β-strands (Fig. 5*D*, bold labels). This disulfide bond retention extends the confident assignment of co-component homologs to include the PsaE/PsaF cassette, and by transitivity to the VtrA/VtrC cassette, given the detected sequence relationship between their interacting partners VtrC and PsaF (*SI Appendix*, Table S1). The more divergent VtrA-like components (Fig. 5*F*) interact with the more divergent VtrC-like components (Fig. 3*B*). The CadC subdomain distances to the rest of the co-component periplasmic domains tend to be less than those of TcpP, and its average similarity to the rest of the structures is higher (Z-score 3.16 for CadC and 1.55 for TcpP). The closest co-component structure to CadC is in the gray zone of homology (*VP3383*; Z-score 6.7). The distance heat map resembles a heat map generated using similarity scores from another structure superposition program (*SI Appendix*, Fig. S2). Although it is tempting to speculate that the co-component signal transduction systems replaced the loss of the CadC helical domain with a gained VtrC-like heterodimer, their structure dissimilarity prevents any confident assignment of homology. This questionable relationship extends to the TcpP component, especially given the tendency of domain recombination among bacterial signaling proteins (6, 7) and the presumed interaction mode of the co-components.

## Discussion

While similarity between the VtrA, ToxR, and other CadC-like HTH domains has long been known (38, 52), sequence divergence in the periplasmic regions has hampered classification. Similarly, while the functional association between the transcription factors and their neighboring gene products is either known or implied (in some cases, the open reading frame is not even annotated), fast sequence evolution of the coactivators has precluded their assignment to known structures or functions. The structure predictions for the VtrA/VtrC-like superfamily suggest that the co-component systems arose from a common two-gene cassette ancestor that has distributed across various enteric bacteria. One of the genes encodes a membrane-tethered transcription factor output domain, and the other encodes a lipid binding–like input cosensor. The VtrA/VtrC co-components function as an obligate heterodimer, where the incomplete barrel for VtrC is unstable. Similarly, the ToxR periplasmic domain is regulated by its stability, which is influenced by both interaction with ToxS and formation of a disulfide (18, 22, 24). Thus, ToxS is thought to activate ToxR by protecting it from degradation (53). Similar chaperone-like mechanisms are proposed for the TcpP/TcpH and PsaE/PsaF co-components (54, 55). While degradation may represent one mode of regulation for these co-component systems, their evolutionary relationship to the VtrA/VtrC bile acid sensor suggests that they may form heterodimers and respond to lipid-like environmental cues.

Enteric bacteria exhibit a dual relationship with bile for survival in the human gastrointestinal tract. On the one hand, both commensal and pathogenic microorganisms must contend with the antimicrobial actions of bile. On the other hand, pathogenic bacteria can use bile as an environmental cue to generate virulence factors (56). The *V. cholerae* master virulence regulator ToxR/ToxS is responsive to bile, yet the detailed mechanism of transcription activation by bile remains elusive (16, 18, 20, 23, 24). A complex regulatory cascade, known as the ToxR/ToxS regulon, ultimately produces the virulence toxin CtxAB and the toxin coregulated pilus TcpA. This complexity confounds an understanding of the contribution of bile to regulation. For example, the downstream ToxR-/ToxS-responsive ToxT regulator represses the regulon in response to arachidonic, linoleic, and oleic acid components of bile, and a structure of ToxT bound to palmitoleic acid supports these observations (57, 58). The ToxR/ToxS regulon is enhanced by other bile constituents. The bile acid taurocholate activates the ToxR/ToxS regulon by promoting TcpP dimerization (59), and ToxR/ToxS itself responds to deoxycholate by increased ToxR–ToxR interactions (23). These data are consistent with TcpH and ToxS sensing bile and activating transcription through their TcpP and ToxR co-component DNA-binding domains.

The relationship between the remaining co-component systems and lipid binding is less clear. The *S. typhimurium* MarT/FidL co-components belong to the Spi-3 pathogenicity island and regulate the expression of their neighboring *MisL* gene. The *MisL* gene product encodes an autotransporter that functions as an intestinal colonization factor (38–40). While this function would be consistent with FidL sensing an environmental cue from the intestine, the mechanism of transcriptional activation by MarT remains to be determined. Interestingly, the *MarT* and *MisL* genes have acquired stop codons and become pseudogenized in the *S. typhimurium* serovar. The loss of MarT/MisL function in the *S. typhimurium* background helped improved virulence in a restricted host cell range (60, 61). Genome comparisons have revealed a distant relationship between the Spi-3 pathogenicity island and the ETT2 type III secretion system in *E. coli* and *Shigella* (44). This relationship identifies *E. coli* K12 and saki YqeI/YqeJ as orthologs of *Salmonella* MarT/FidL. The corresponding gene region is missing in the incomplete ETT2 gene cluster from *Shigella*, although a paralogous uncharacterized co-component cassette exists elsewhere in the *S. flexneri* genome (*SF3508*/*SF3507*). The *E. coli* K12 *YqeI*/*YqeJ* cassette also appears to be a remnant of the complete ETT2 pathogenicity island present in pathogenic strains like *E. coli* O157:H7 saki. Pathogenic *E. coli* O157:H7 saki includes a paralogous *YqeI*/*YqeJ* gene cassette (*GrvA/FidL*) that activates the LEE-dependent adherence (41), and many of the LEE adherence genes are differentially regulated by the presence of bile acids (62).

The similarity in folds between the CadC N-terminal periplasmic domain and the periplasmic domains from the co-component transcription factors points toward their derivation from a common membrane-tethered transcription factor. The Cad system, which is widespread in enteric bacteria, helps maintain a neutral cytoplasmic pH in the acidic environment of the stomach (10, 12). The CadC transcription factor is activated in part by acid conditions, which reduce the N-terminal periplasmic domain disulfide (63). Some of the co-component transcription factors such as ToxR are also known to respond to pH. However, ToxR is activated in alkaline conditions and requires oxidized disulfides (23). Although the positions of disulfides in the periplasmic domains of these transcription factors are not preserved (Fig. 5*E*), they may point to a common function in pH sensing. In fact, a disulfide stabilizes the unusual C-terminal strand topology of TcpP. Low pH may reduce the disulfide and release the strand to allow interaction with TcpH. In fact, the solution structure of oxidized ToxR revealed a similar structure instability in the C terminus, where the periplasmic domain presumably interacts with its ToxS partner. Such structure plasticity may help explain the fast evolution of the periplasmic domain, as the unstructured protein regions can evolve independent of their primary sequence if they maintain the disulfide. Apart from VtrA, which lacks a disulfide, the co-component transcription factors maintain a disulfide Cys residue near the C terminus (Fig. 5*E*). This position tends to correspond with the sequence overlap from the neighboring gene (*SI Appendix*, Fig. S3), providing a mechanism for its preservation.

Overall, our studies support a model for a signaling paradigm used by enteric bacteria to sense extracellular cues in the periplasm. The co-component signal transduction system includes an input periplasmic sensor that is tightly associated with its transcription factor, as exhibited by the operon organization, coevolution, and obligate periplasmic domain interaction. This co-component system provides an additional, albeit specialized, mechanism for transmitting an extracellular signal sensed by a periplasmic input through the membrane to a transcription factor output domain, adding to the canonical transmembrane one-component and two-component signaling systems (Fig. 1). Understanding the mechanism of signal transduction at the membrane and the ligands or conditions that activate these co-components will provide seminal information on how bacteria respond to their environment.

## Methods

### Extending the VtrA/VtrC Co-component Superfamily.

Sequences corresponding to *V. parahaemolyticus* ToxR (BAC59083.1) and VtrA (BAC62675.1) were used as queries with PSI-BLAST (64) (five iterations, E-value cutoff 0.001) to search against a protein sequence database comprised of bacterial reference genomes. The database was constructed using makeblastdb with protein sequences from RefSeq reference genomes defined by the National Center for Biotechnology Information (NCBI) (15 genomes) together with protein sequences from the *V. parahaemoliticus* RIMD, *V. cholerae* MS6, and *Yersinia pestis* A1122 genomes with gene accessions from the NCBI (*SI Appendix*, Table S2). Identified VtrA-related hits were filtered for a predicted TMH using Phobius (65). The genome neighborhoods of candidate TMH-containing VtrA-like sequences (34 genes) were inspected for a tandem operon of the VtrA-like sequence and a downstream potential VtrC-like sequence with a predicted N-terminal TMH or signal peptide (SP). To account for potential overlapping open reading frames (ORFs) (as is the case for *VtrA*/*VtrC*), the upstream in-frame region was translated for cases where the adjacent gene was missing a predicted N-terminal TMH or SP.

### VtrC-Like Sequence Clustering and Family-Level Conservations.

For clustering VtrC-like sequences, close sequence homologs of each of the VtrC-like family members with a predicted lipid binding domain were used as queries to search the RefSeq Select database using the NCBI server (default values, search until convergence, or relaxed E-value cutoff 0.02 for small families). All sequences were clustered with CLANS (66) using BLAST scores (E-value cutoff 0.01). Identified sequences were submitted to the MAFFT server (67) to generate multiple sequence alignments with the default strategy. Residue conservations were mapped to the B-factors of the VtrC structure (5kew, chain D) or the structure models of ToxS and FidL using Al2Co (68) for visualization in PyMOL with a rainbow color scale from blue (variable) to red (conserved).

### Single and Complex Structure Prediction.

Candidate VtrC-like sequences found in tandem with VtrA-like transmembrane transcription factors were submitted to AlphaFold2 (32) structure prediction using ColabFold (35), which replaces the homology detection of AlphaFold2 with MMseqs2 (69), or with a local adaptation of AlphaFold described three paragraphs below.

Starting from candidate co-component protein pairs, we searched for the homologs of each protein encoded by nucleotide sequences in the European Nucleotide Archive database and the Integrated Microbial Genomes and Microbiomes database of the Joint Genome Institute. We used six rounds of iterative HMMER (70) search with E-value cutoffs of 10 to 12, 10 to 12, 10 to 12, 10 to 12, 10 to 6, and 10 to 3, respectively. Homologs found in each round of sequence search were used to construct the sequence profile for each protein using HMMER hmmbuild, which was used to identify more homologs in the next round. We filtered the homologs found in the last round of database search by their coverage (> 60%) over the query sequence and recorded their loci on the nucleotide sequences.

For each protein pair, their homologs that are encoded next to each other in the nucleotide sequences were extracted. The sequences of these protein pairs were concatenated, and the multiple sequence alignment (MSA) was derived from the pairwise sequence alignments made by HMMER. The MSA was then filtered by sequence identity (maximal identity for remaining sequences ≤ 90%) and gap ratio in each sequence (maximal gap ratio ≤ 50%), and the resulting nonredundant MSA was used as input for complex structure modeling.

We deployed AlphaFold (32), a method designed to model protein monomers, to model protein complexes using two modifications: 1) we provided the concatenated alignments of protein pairs (described in the paragraph above) to AlphaFold instead of sequence alignments for single proteins, and 2) we changed the positional encoding used in AlphaFold to represent a chain break between two proteins. The relative positional encoding was calculated based on residue numbers and was the only input that encoded chain connectivity in AlphaFold. We added 200 to the residue numbers of the second protein in each concatenated alignment to let the AlphaFold network know that there was no chain connection between the two proteins. This simple modification enabled us to model a large set of eukaryotic protein complexes (48), and it is expected to produce a high-quality model for candidate one-component signal transduction systems with enough homologs (∼100).

### VtrA-Like Periplasmic Domain Heat Map.

The experimental structures corresponding to the VtrA periplasmic domain (5kewA), the ToxR periplasmic domain (6uueA), and the CadC N-terminal periplasmic domain (3lyaA, residues 194 to 316) were compared to the models for GrvA (189-270), YqeI (186-269), STM0344 (170-247), SF3808 (170-254), BprP (256-326), PsaE (160-214), YP3564 (162-219), YP3383 (235-348), and TcpH (169-221). All-against-all structures were compared using DaliLte (71). Pairwise (Z_AB_) and self (Z_AA_, Z_BB_) Z-scores were transformed into distances using the following equation: −ln(Z_AB_/[minimum of Z_AA_,Z_BB_]).

## Data Availability

All study data are included in the article and/or *SI Appendix*.
